# Supplementary material for: Pea Albumin Attenuates Dextran Sulfate Sodium-Induced Colitis by Regulating NF-κB Signaling and the Intestinal Microbiota in Mice
Source: Nutrients. 2022 Sep 1;14(17):3611. doi: 10.3390/nu14173611 (PMC9460122; doi:10.3390/nu14173611)
Supplement: Supplementary file 1 [file nutrients-14-03611-s001.zip › nutrients-1761393-supplementary.pdf]

Online Supporting Material

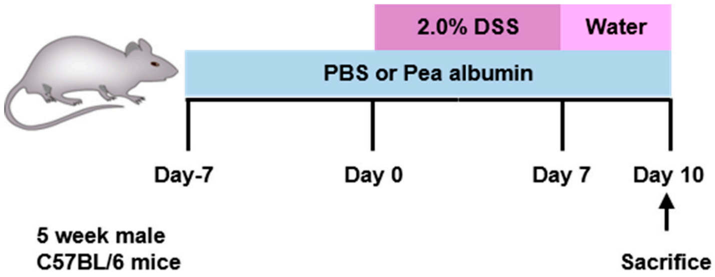

Supplemental Figure S1 Scheme of the animal experimental design. DSS, dextran sulfate sodium.

Online Supporting Material

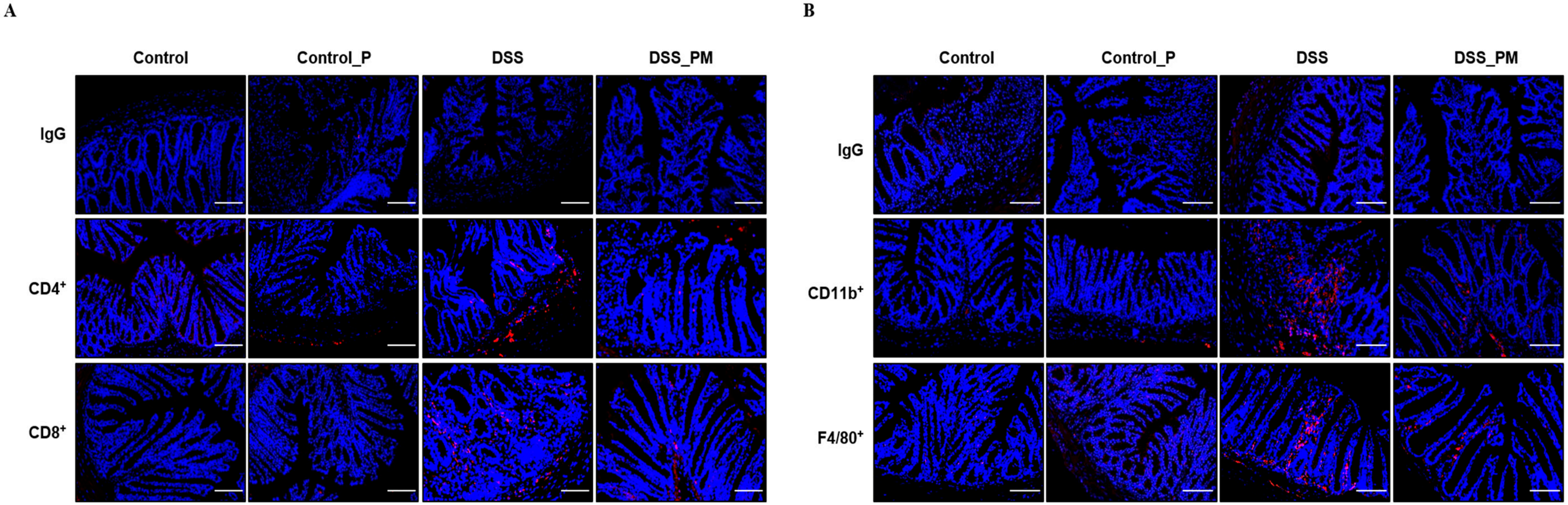

Supplemental Figure S2 (A, B) Representative immunofluorescent staining image of $CD4^+$, $CD8^+$, $CD11b^+$, and $F4/80^+$ cells (red). Nuclei were stained with Hoechst 33342 (blue). Scale bar means 100 μm. Data are shown as mean ± SEM. n = 9 per group. DSS, dextran sulfate sodium; Control_P, 0.75 g/kg body weight pea albumin by oral gavage; DSS_PM, 2.0% w/v DSS in drinking water + 0.75 g/kg body weight pea albumin by oral gavage.

Online Supporting Material

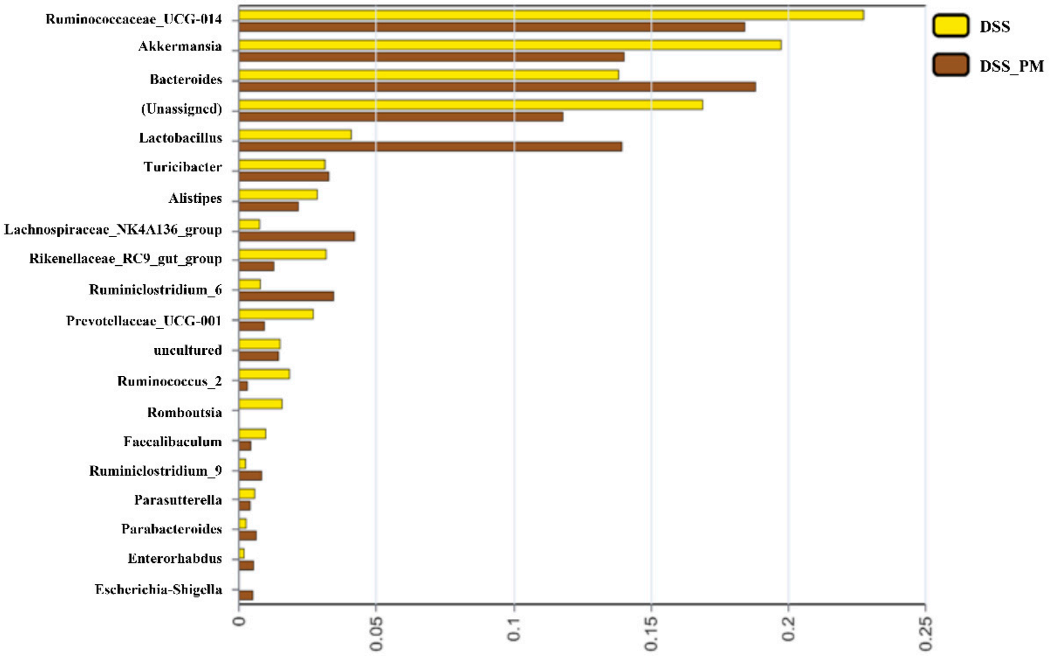

Supplemental Figure S3 The relative abundance of the gut microbiota between DSS group and DSS_PM group. DSS, dextran sulfate sodium; DSS_PM, 2.0% wt/vol + 0.75 g/kg body weight pea albumin.

Online Supporting Material

Supplemental Table S1 Parameters and criteria of histological damage evaluation

Parameters	Score	Histological features
	0	No change
(1) Loss of epithelial surface	1	Localized and mild
(2) Destruction of crypt	2	Localized and moderate
(3) Infiltration of inflammatory cells	3	Localized and severe
	4	Extensive and moderate
	5	Extensive and severe

[1] Histological score was the sum of scoring from parameter (1), (2), and (3).

[2] Adapted from Nishiyama et al. with modifications.

Online Supporting Material

Supplemental Table S2 Primer sequence for real-time PCR

Genes	Forward primer (5' to 3')	Reverse primer (5' to 3')
TNF-α	TGAGGTCAATCTGCCCAAGT	GGGGTCAGAGTAAAGGGGTC
IFN-γ	GAGAGGCCCTATCCCAACTC	TCAAGAGAGTAGGGAGGGCT
IL-1β	CAGGCAGGCAGTATCACTCA	TGTCCTCATCCTGGAAGGTC
IL-6	CTGCAAGAGACTTCCATCCAG	AGTGGTATAGACAGGTCTGTT
IL-17A	GAAGGCCCTCAGACTACCTC	CTTTCCCTCCGCATTGACAC
IL-22	GACAGGTTCCAGCCCTACAT	TCGCCTTGATCTCTCCACTC
GAPDH	AAGCCCATCACCATCTTCCA	CACCAGTAGACTCCACGACA

Reference

Nishiyama, Y., Kataoka, T., Yamato, K., Taguchi, T., Yamaoka, K., Suppression of dextran sulfate sodium-induced colitis in mice by radon inhalation. *Mediators of inflammation* 2012, *2012*, 239617.
